# Deletion of the Viral Anti-Apoptotic Gene *F1L* in the HIV/AIDS Vaccine Candidate MVA-C Enhances Immune Responses against HIV-1 Antigens

**DOI:** 10.1371/journal.pone.0048524

**Published:** 2012-10-31

**Authors:** Beatriz Perdiguero, Carmen Elena Gómez, Jose Luis Nájera, Carlos Oscar S. Sorzano, Julie Delaloye, Rubén González-Sanz, Victoria Jiménez, Thierry Roger, Thierry Calandra, Giuseppe Pantaleo, Mariano Esteban

**Affiliations:** 1 Department of Molecular and Cellular Biology, Centro Nacional de Biotecnología, Consejo Superior de Investigaciones Científicas (CSIC), Madrid, Spain; 2 Biocomputing Unit, Centro Nacional de Biotecnología, Consejo Superior de Investigaciones Científicas (CSIC), Madrid, Spain; 3 Infectious Diseases Service, Department of Medicine, Centre Hospitalier Universitaire Vaudois and University of Lausanne, Lausanne, Switzerland; 4 Division of Immunology and Allergy, Centre Hospitalier Universitaire Vaudois, Lausanne, Switzerland; Mayo Clinic, United States of America

## Abstract

Vaccinia virus (VACV) encodes an anti-apoptotic Bcl-2-like protein F1 that acts as an inhibitor of caspase-9 and of the Bak/Bax checkpoint but the role of this gene in immune responses is not known. Because dendritic cells that have phagocytosed apoptotic infected cells cross-present viral antigens to cytotoxic T cells inducing an antigen-specific immunity, we hypothesized that deletion of the viral anti-apoptotic *F1L* gene might have a profound effect on the capacity of poxvirus vectors to activate specific immune responses to virus-expressed recombinant antigens. This has been tested in a mouse model with an *F1L* deletion mutant of the HIV/AIDS vaccine candidate MVA-C that expresses Env and Gag-Pol-Nef antigens (MVA-C-ΔF1L). The viral gene *F1L* is not required for virus replication in cultured cells and its deletion in MVA-C induces extensive apoptosis and expression of immunomodulatory genes in infected cells. Analysis of the immune responses induced in BALB/c mice after DNA prime/MVA boost revealed that, in comparison with parental MVA-C, the mutant MVA-C-ΔF1L improves the magnitude of the HIV-1-specific CD8 T cell adaptive immune responses and impacts on the CD8 T cell memory phase by enhancing the magnitude of the response, reducing the contraction phase and changing the memory differentiation pattern. These findings reveal the immunomodulatory role of *F1L* and that the loss of this gene is a valid strategy for the optimization of MVA as vaccine vector.

## Introduction

The search for a safe and effective HIV vaccine able to induce long-lived protective immunity has stimulated the development of recombinant live vaccine candidates with good safety and immunogenicity profiles. The recent Thai phase III clinical trial (RV144) combining, in a prime-boost strategy, the recombinant poxvirus vector ALVAC and the protein gp120 and showing a 31.2% of protection against HIV infection [1], has raised considerable interest in the use of improved attenuated poxvirus recombinants as HIV vaccine candidates. Among poxviruses, recombinants based on the highly attenuated strain MVA expressing different HIV-1 antigens have been extensively studied in preclinical [2], [3], [4] and clinical trials with encouraging results [5], [6], [7], [8], [9], [10], [11], [12].

MVA was derived from the Ankara strain of vaccinia virus (VACV) after more than 500 passages on chicken embryo fibroblast cells. During this extensive passage, six regions (approximately 31 kb) were lost from the viral genome, resulting in the deletion of a number of host-range restriction and immunomodulatory genes [13], [14]. As a result of the deletion of host-range restriction genes, replication of MVA in most non-avian cell types aborts at a late stage of the virus life cycle [15], [16]. MVA provides a high level of gene expression and triggers strong immune responses when delivering foreign antigens in animals and humans [4], [17], [18], [19]. However, further improvement of MVA-based vaccines with enhanced magnitude, breadth, polyfunctionality and durability of the immune response is needed.

Virus detection by the infected cell often results in the induction of apoptosis as an anti-viral mechanism to limit viral spread. For this reason, viruses have evolved strategies that target key components of the apoptotic cascade, including inhibitors of the intrinsic pathway of apoptosis [20], [21], [22], [23]. Apoptosis is a complex and highly regulated mechanism of programmed cell death that is mediated by a family of cysteine proteases, or caspases, whose activation is triggered by a number of external or cellular signals [24], [25], [26]. *In vivo*, the initial non-specific anti-viral effect of apoptosis induction can be potentiated by the capture of apoptotic bodies by dendritic cells (DCs). This uptake and processing of apoptotic infected cells by DCs can enhance MHC class I presentation of viral antigens and increase the immune response, phenomenon known as cross-presentation [27]. In this context, the induction of apoptosis by deletion of viral anti-apoptotic genes in recombinant MVA expressing heterologous antigens could be a strategy to enhance the immunogenicity against a vaccine antigen.

Vaccinia virus (VACV), the prototypic member of the *Poxviridae* family, expresses a mitochondrial-associated inhibitor of apoptosis encoded by *F1L* gene. The *F1L* open reading frame in VACV strain Copenhagen encodes a tail-anchored protein of 226 amino acids that localizes to the outer mitochondrial membrane, where it inhibits the loss of the inner mitochondrial membrane potential and the release of cytochrome *c* in response to a wide variety of apoptotic stimuli [28], [29], [30]. Its C-terminal region contains a hydrophobic 12-amino acid transmembrane domain flanked by positively charged lysines followed by an 8-amino acid hydrophilic tail, which are necessary for mitochondrial targeting as well as for the anti-apoptotic function [29]. The *F1L* open reading frame is highly conserved between the MVA genome (ORF 029) and VACV strain Copenhagen (98% amino acid identity) [13]. The anti-apoptotic mechanism of action of F1 has been extensively studied. The success of the apoptosis induced by the mitochondrial pathway depends on the balance between pro- and anti-apoptotic members of the Bcl-2 (B-cell lymphoma-2) family of proteins, which are typically characterized as containing one or more Bcl-2 homology (BH) domains [31]. F1 has been reported to interact with the BH3 domain of the pro-apoptotic protein Bak, inhibiting tBid-induced Bak activation [30], [32]. This interaction is mediated by highly divergent BH domains in F1 [33] that were verified by the crystal structure of F1 protein from MVA strain [34]. This structure confirmed that despite a lack of apparent sequence homology to Bcl-2 proteins, F1 adopts a Bcl-2-like fold. In addition to interacting with Bak, F1 has also been reported to associate with the BH3-only protein Bim indirectly inhibiting the activation of the pro-apoptotic protein Bax following an apoptotic stimulus [35], [36]. In this context, it has been proposed that F1 orthologs represent the only orthopoxvirus Bcl-2-like proteins to directly inhibit the Bak/Bax checkpoint [37]. F1 has also been reported to bind to and inhibit caspase-9, the apical protease in the intrinsic (mitochondrial) pathway, independent of its interactions with pro-apoptotic Bcl-2 family proteins [38]. An N-terminal α helix of F1 preceding the Bcl-2-like fold is responsible for this caspase-9 binding and inhibition [38], [39]. Therefore, F1 is the first viral anti-apoptotic protein described that functions both as a suppressor of pro-apoptotic Bcl-2 family proteins and as an inhibitor of caspase-9, thereby blocking two sequential steps in the mitochondrial apoptotic pathway. A molecular framework of how cells detect MVA infection *in vitro* and induce apoptosis and how the virus blocks apoptosis by interfering with critical steps of its signal transduction has been previously reported using either MVA wt or *F1L* deletion mutant [36], [40].

Because MVA infection of different human cell lines induces apoptosis and cross-presentation by DCs [41] and MVA recombinants expressing HIV antigens have shown good immunogenicity profiles in animal models and in humans [42], [43], in the present study we have asked to what extent enhanced apoptosis induction by deletion of *F1L* gene can improve the capacity of MVA-C, an attenuated poxvirus vector expressing HIV-1 Env and Gag-Pol-Nef antigens from clade C [44], to activate specific adaptive and memory immune responses to HIV-1 antigens in a BALB/c mouse model.

## Results

### Generation and *in vitro* Characterization of MVA-C-ΔF1L Deletion Mutant

**Figure 1:**
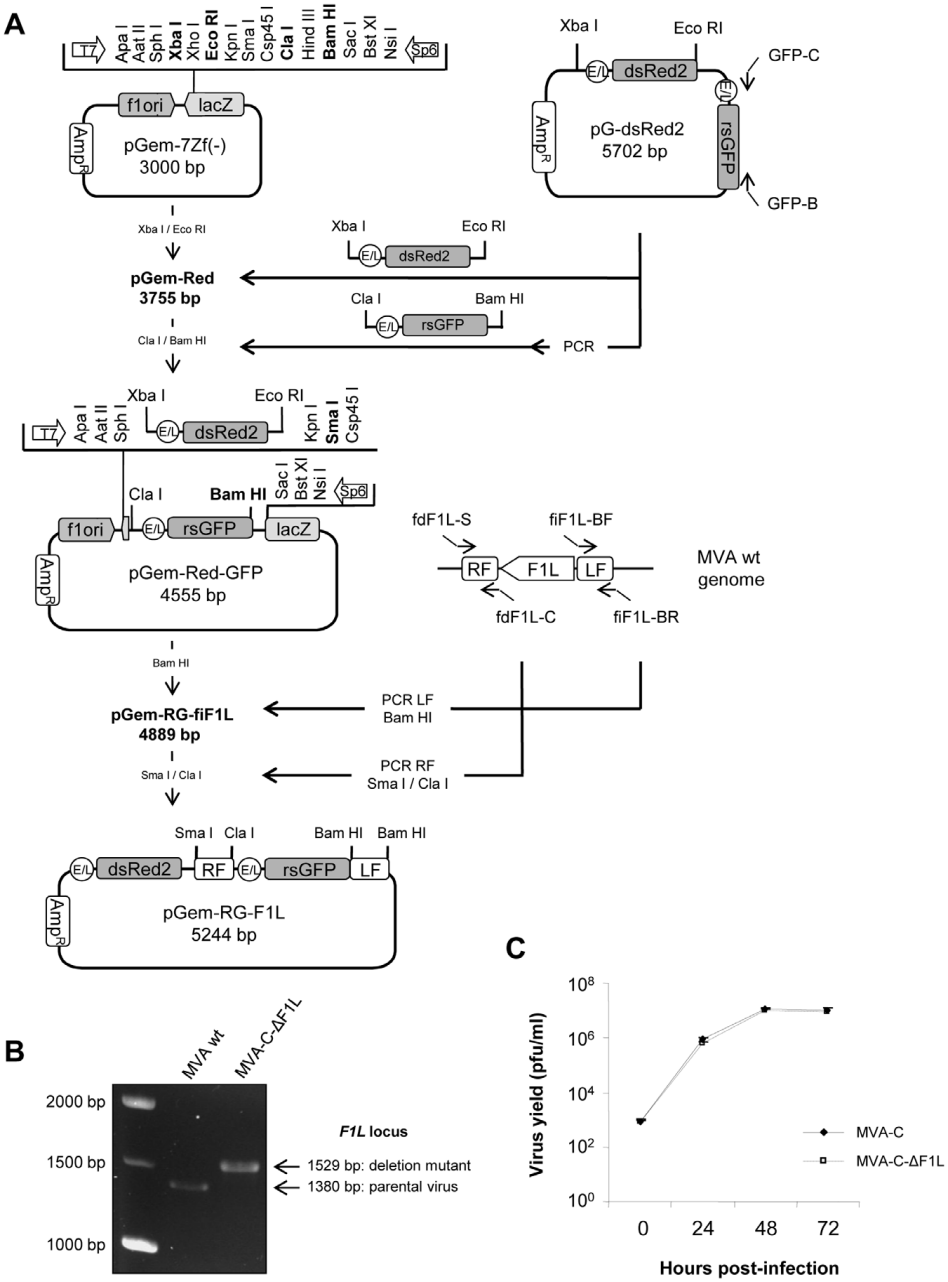
Generation and *in vitro* characterization of MVA-C-ΔF1L deletion mutant. (A) Scheme of construction of the plasmid transfer vector pGem-RG-F1L. The plasmid transfer vector pGem-RG-F1L was obtained by sequential cloning of markers dsRed2 and rsGFP and *F1L* recombination flanking sequences into the plasmid pGem-7Zf. The dsRed2 gene under the control of the synthetic early/late (E/L) promoter was excised from plasmid pG-dsRed2 and inserted into pGem-7Zf to generate pGem-Red. The rsGFP gene under the control of the synthetic E/L promoter was amplified by PCR from plasmid pG-dsRed2, digested and inserted into the plasmid pGem-Red to generate pGem-Red-GFP. MVA genome was used as template to amplify the left flanking sequence of *F1L* gene. The PCR product was digested and inserted into pGem-Red-GFP previously digested and dephosphorylated by incubation with Calf intestinal Alkaline Phosphatase (CIP) to generate the plasmid pGem-RG-fiF1L. The right flanking sequence of *F1L* gene was amplified by PCR from MVA genome. The PCR product was digested and inserted into plasmid pGem-RG-fiF1L to generate the plasmid transfer vector pGem-RG-F1L. (B) Confirmation of *F1L* gene deletion by PCR analysis. Viral DNA was extracted from DF-1 cells infected with MVA wt or MVA-C-ΔF1L at 5 PFU/cell. Primers fdF1L-S and fiF1L-BR spanning *F1L* flanking sequences were used for PCR analysis of *F1L* locus. In parental MVA, a 1380 bp-product is obtained while in deletion mutant a unique 1529 bp-product is observed. (C) Analysis of virus growth of MVA-C-ΔF1L in CEF cells. Monolayers of CEF cells were infected with MVA-C or MVA-C-ΔF1L at 0.01 PFU/cell. At different times post-infection (0, 24, 48 and 72 hours), cells were collected and infectious viruses were quantified by immunostaining assay.

MVA-C-ΔF1L deletion mutant, in which *F1L* ORF has been substituted for a rsGFP expression cassette, was generated as described under Materials and Methods using as parental virus the recombinant MVA-C that expresses the HIV-1 Env and Gag-Pol-Nef antigens from clade C [44]. The correct deletion of *F1L* was confirmed by PCR using primers annealing in *F1L* flanking sequences. As shown in Figure 1B, *F* *1L* ORF has been successfully deleted and substituted for the rsGFP expression cassette and no wild-type contamination was present in MVA-C-ΔF1L preparation. To determine if *F1L* gene affects virus replication in cell culture, we compared the growth kinetics of MVA-C-ΔF1L deletion mutant with its parental virus MVA-C in CEF cells. Figure 1C shows that the kinetics of growth were similar between parental and *F1L* deletion mutant. This indicates that *F1L* gene is not required for virus replication in cultured cells and its deletion does not affect virus growth kinetics.

### MVA-C-ΔF1L Deletion Mutant Expresses HIV-1 Antigens in a Stable Manner

**Figure 2:**
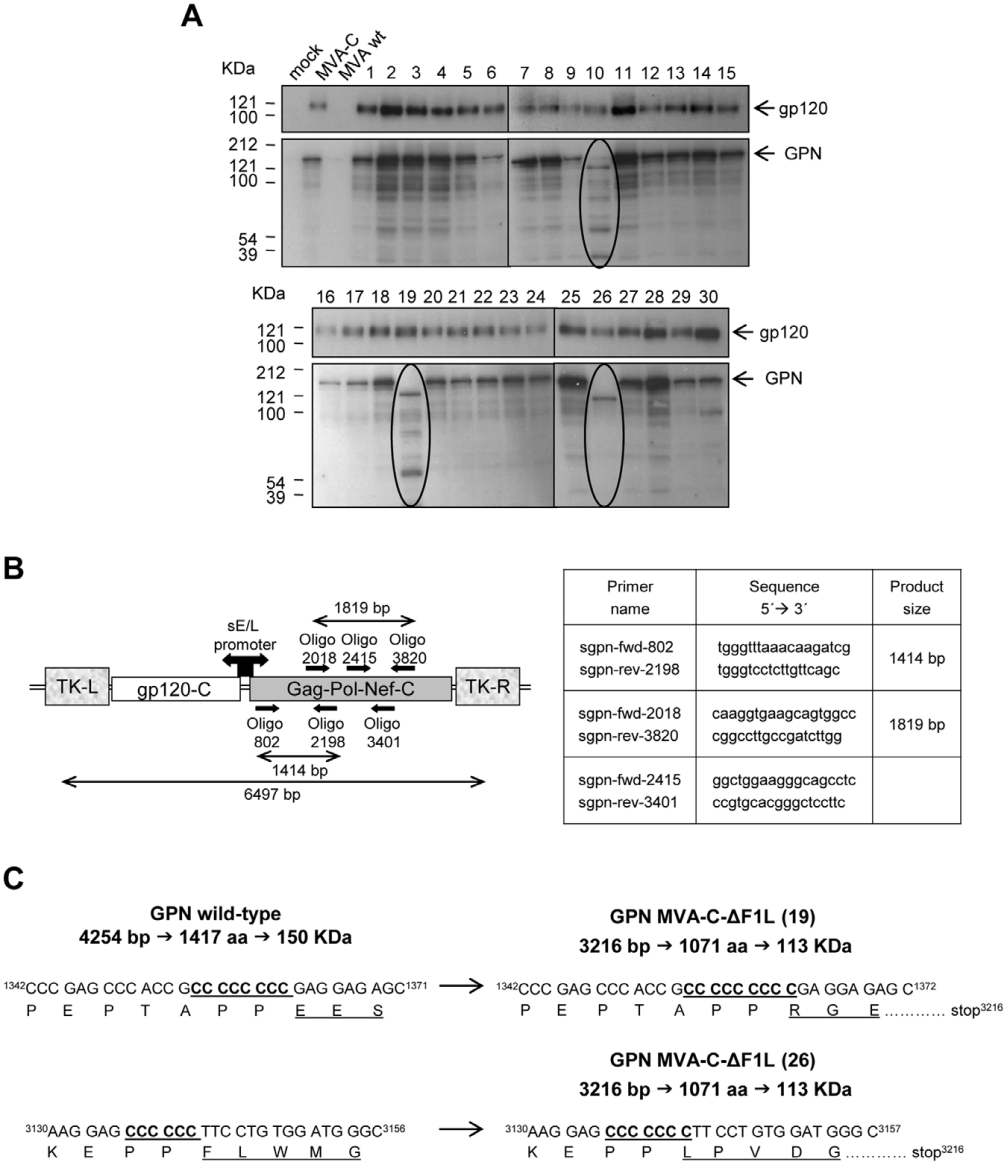
Analysis of the stability of gp120 and GPN proteins expressed by MVA-C-ΔF1L. (A) Thirty individual plaques from MVA-C-ΔF1L were grown in DF-1 cells, lysed, proteins fractionated by SDS-PAGE and analyzed by Western-blot with specific antibodies. The expression of gp120 or GPN proteins in mock-infected cells or cells infected with MVA wt, MVA-C or with individual plaques from MVA-C-ΔF1L (1–30) is shown. Arrows indicate the correct size of gp120 and GPN proteins. (B) Schematic representation of the HIV-1 inserts within the TK viral locus of MVA-C. The positions and sequences of the different sets of primers used for PCR analysis and sequencing of GPN polyprotein and the expected sizes of PCR products are represented. (C) Identification of mutations of GPN polyprotein. Viral DNA extracted from DF-1 cells infected with plaques 19 or 26 of MVA-C-ΔF1L was used as template to amplify and sequence different regions of GPN polyprotein. In plaque 19, one cytosine insertion was identified at position 1355–1362 producing a frame-shift and a premature stop codon at position 3216. In plaque 26, one cytosine insertion was identified at position 3136–3141 producing a frame-shift and a premature stop codon at position 3216.

When considering the use of a recombinant vector as a vaccine, it is critical to ensure that recombinants can be grown to large scale without loss of the transgene. Therefore, a stability test was performed. Monolayers of DF-1 cells were infected with MVA-C-ΔF1L at passage 11 and 30 individual plaques were picked up, grown and analyzed by Western-blot with specific anti-gp120 or anti-p24 antibodies. As shown in Figure 2A, out of 30 isolated plaques none of them represent wild-type reversion. All (100%) express the correct full-length gp120 protein and for Gag-Pol-Nef (GPN) polyprotein, 27 (90%) express the correct full-length polyprotein and 3 (10%) express a truncated form. Similar percentages were obtained with the parental virus MVA-C (data not shown). These results reveal that MVA-C-ΔF1L deletion mutant is genetically stable and that gp120 is slightly more stable than GPN.

To identify the mutations present in truncated forms of GPN polyprotein, viral DNA extracted from DF-1 cells infected with plaques 19 or 26 of MVA-C-ΔF1L was used as template to amplify and sequence different regions of the polyprotein (Figure 2B). Plaque 10 was not analyzed because it showed the same expression pattern than plaque 19. In the case of plaque 19, oligonucleotides sgpn-fwd-802 and sgpn-rev-2198 were used to amplify a 1414 bp-product comprising from positions 802 to 2198 of the entire 4254 bp sequence of GPN. This PCR product was sequenced with the same primers used for PCR amplification and, as shown in Figure 2C, an additional cytosine was inserted in a region of 8 consecutive cytosines at position 1355–1362. Wild-type GPN sequence comprises 4254 bp encoding a polyprotein of 1417 residues and 150 KDa of molecular weight. In the case of plaque 19, the additional cytosine produces a frame-shift and a premature stop codon at position 3216, encoding a polyprotein of 1071 residues and 113 KDa of molecular weight. In the case of plaque 26, oligonucleotides sgpn-fwd-2018 and sgpn-rev-3820 were used to amplify a 1819 bp-product comprising from positions 2018 to 3820 of the entire 4254 bp sequence of GPN. This PCR product was sequenced with primers sgpn-fwd-2415 and sgpn-rev-3401 and an additional cytosine was inserted in a region of 6 consecutive cytosines at position 3136–3141 (Figure 2C). This additional cytosine generates a frame-shift and a premature stop codon at position 3216, producing a polyprotein of 1071 residues and 113 KDa of molecular weight. As shown in Figure S1, GPN sequence contains cytosine-rich regions. It comprises 14 regions of 5 consecutive cytosines, 1 region of 8 consecutive cytosines (cytosine insertion detected in plaque 19) and 1 region of 6 consecutive cytosines (cytosine insertion detected in plaque 26). Probably, these cytosine-rich regions serve as hot spots for spontaneous point mutations [45], turning GPN into less stable protein than gp120, which lacks cytosine-rich regions. Nonetheless, the insertion cassette (gp120-GPN) placed in the TK locus was highly stable within the MVA genome.

### Apoptosis Induction by MVA-C-ΔF1L Deletion Mutant

**Figure 3:**
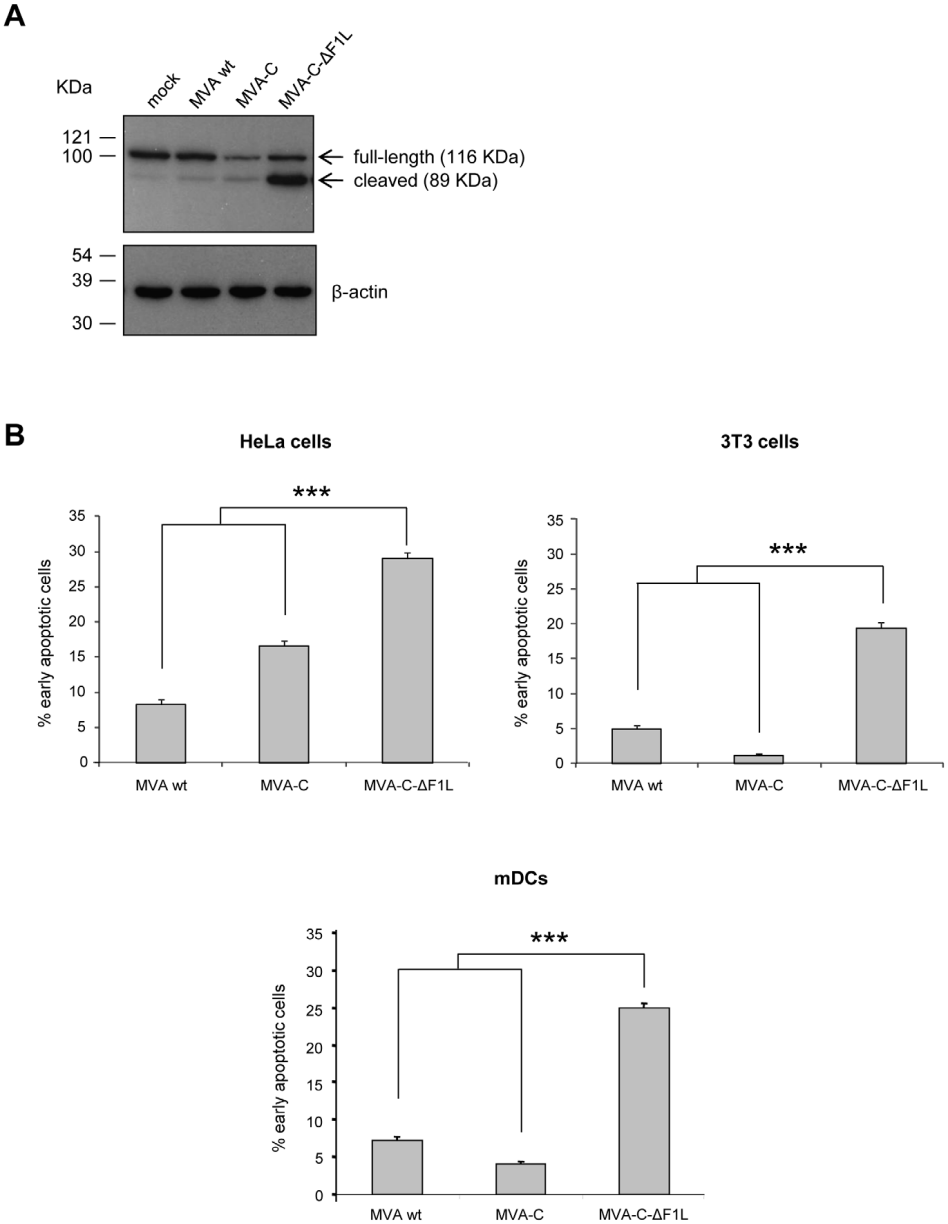
Analysis of apoptosis induced by MVA-C-ΔF1L deletion mutant. (A) PARP cleavage was analyzed by Western-blot in HeLa cells mock-infected or infected with MVA wt, MVA-C or MVA-C-ΔF1L at 5 PFU/cell for 16 hours. Detection of cellular β-actin protein was used as an internal loading control. (B) Annexin V binding assay of HeLa, 3T3 cells or murine DCs infected with MVA wt, MVA-C or MVA-C-ΔF1L at 5 PFU/cell. At 16 hours post-infection, the infected cells were stained with Annexin V and propidium iodide as described under Materials and Methods and the percentages of early apoptotic cells (Annexin V positive, PI negative) were determined by flow cytometry. All data are mock-infected cells-subtracted. HeLa or 3T3 cells treated with staurosporine (0.5 µM) were used as positive control (not shown). *** *p*<0.001. *p* value indicates significantly higher response compared to MVA wt or MVA-C-infected cells.

If the role of F1 is to block apoptosis triggered in the cell as a response to VACV infection, the deletion mutant MVA-C-ΔF1L would be expected to induce apoptosis. To confirm this, we analyzed the PARP cleavage by Western-blot and the integrity of the cell membrane by Annexin V binding assay. The cleavage of PARP [poly (ADP-ribose) polymerase] commonly occurs in apoptotic cells by the activation of caspases. In human PARP, the cleavage separates PARP´s amino-terminal DNA binding domain (24 KDa) from its carboxy-terminal catalytic domain (89 KDa) [46]. Therefore, PARP cleavage serves as a marker of cells undergoing apoptosis and was analyzed by Western-blot in HeLa cells mock-infected or infected with MVA wt, MVA-C or MVA-C-ΔF1L (5 PFU/cell) at 16 hours post-infection. The antibody used recognizes both forms of the protein, full-length and cleaved PARP. As shown in Figure 3A, the 116-KDa full-length PARP was mostly cleaved (89 KDa) in cells infected with MVA-C-ΔF1L while in cells mock-infected or infected with MVA wt or MVA-C this cleavage was reduced.

Changes in the asymmetry of the cell membrane associated with the externalization of phosphatidylserine (PS) are one of the events observed in a cell undergoing apoptosis. Annexin V has high affinity to PS so, together with propidium iodide (PI), it is a useful tool to quantify apoptosis by flow cytometry. This assay allows to determine the percentage of viable cells (both Annexin V and PI negative), early apoptotic cells (Annexin V positive, PI negative) and cells that are in late apoptosis or already dead by apoptotic or necrotic pathways (both Annexin V and PI positive). Thus, HeLa or 3T3 cells mock-infected or infected with MVA wt, MVA-C or MVA-C-ΔF1L at 5 PFU/cell were stained with Annexin V and propidium iodide at 16 hours post-infection as described under Materials and Methods. As this analysis does not distinguish between cells that have undergone apoptosis versus those that have died as a result of a necrotic pathway, we have determined the percentage of early apoptotic cells (Annexin V positive, PI negative). As shown in Figure 3B (upper panels), 29% (HeLa) or 19% (3T3) of cells infected with MVA-C-ΔF1L were Annexin V positive^/^PI negative, which represents a significant increase with respect to MVA-C-infected cells (HeLa: 17%; 3T3∶1%) (*p*<0.001). The percentage of total death (Annexin V and PI positive cells) was also enhanced in MVA-C-ΔF1L-infected HeLa or 3T3 cells (data not shown). Finally, we wanted to confirm this enhanced apoptosis by *F1L* deletion in a more physiological system so we performed the Annexin V/PI assay in mock-infected or MVA wt, MVA-C or MVA-C-ΔF1L-infected murine bone marrow-derived dendritic cells (mDCs) at 5 PFU/cell for 16 hours. As shown in Figure 3 (lower panel), the percentage of early apoptotic cells is significantly increased in the case of MVA-C-ΔF1L-infected mDCs (25%) compared with the percentages obtained in MVA wt (7%) or MVA-C (4%)-infected mDCs (*p*<0.001), as previously observed for the case of HeLa and 3T3 cell lines. These results indicate that *F1L* deletion from MVA markedly increases the levels of apoptosis.

### MVA-C-ΔF1L Up-regulates Type I IFN and Pro-inflammatory Cytokine Expression in Murine Macrophages and Dendritic Cells

**Figure 4:**
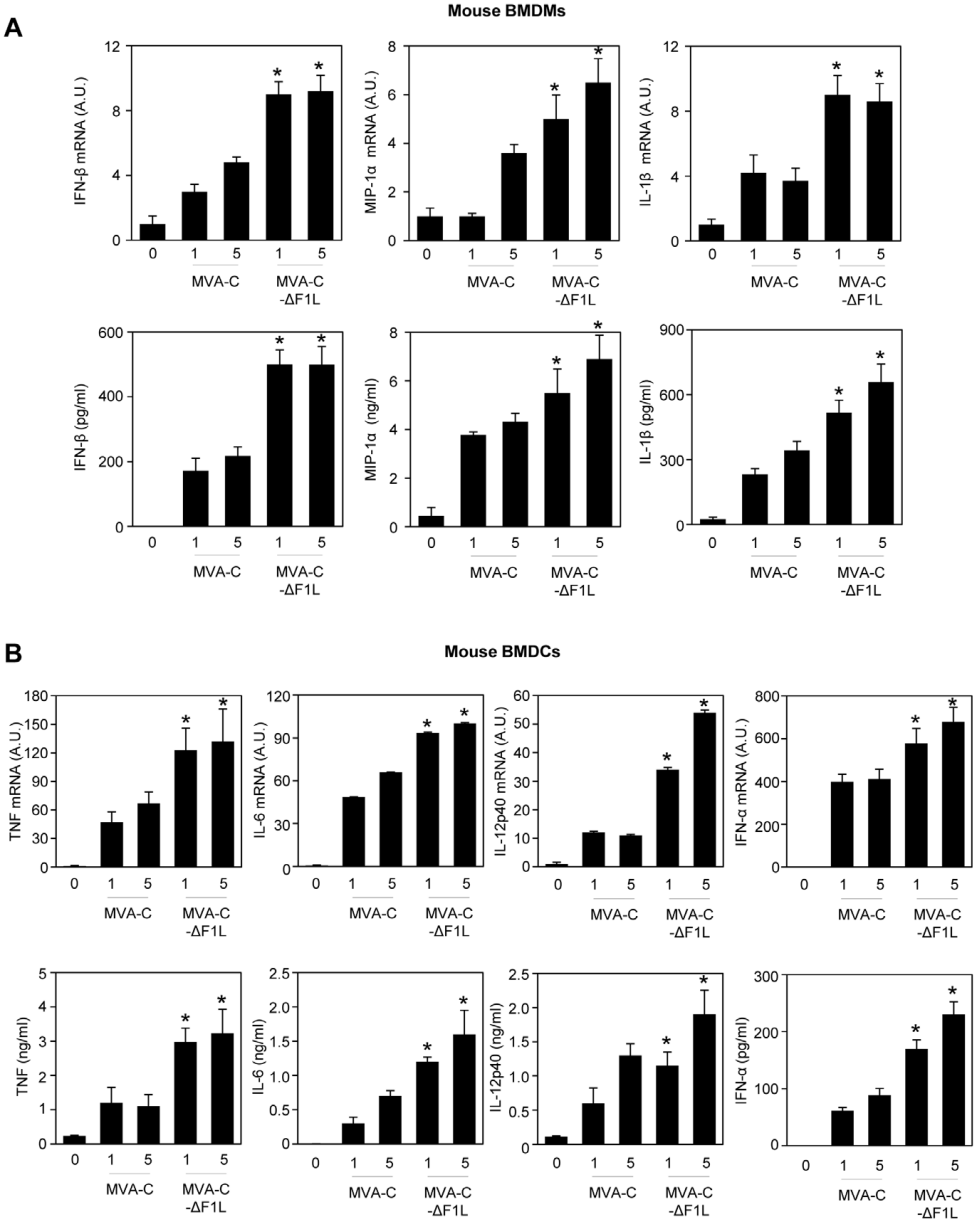
MVA-C-ΔF1L deletion mutant enhances the production of type I IFNs and cytokines in murine macrophages (A) and dendritic cells (B). BMDMs or BMDCs were mock-infected (0) or infected with MVA-C or MVA-C-ΔF1L at 1 or 5 PFU/cell for 6 hours (upper panels) or 24 hours (lower panels). IFN-α, IFN-β, IL-1β, TNF, IL-6, IL-12p40 and MIP-1α mRNA (upper panels) and protein (lower panels) levels were quantified by RT-PCR and ELISA, respectively. mRNA results are expressed as the ratio of the gene of interest to *Hprt* mRNA levels. A.U: arbitrary units. ELISA results are means ± SD of duplicate samples from one experiment and are representative of two experiments. * *p*<0.05 for all conditions comparing MVA-C-ΔF1L to MVA-C at the same MOI.

Since mitochondria are not only involved in the control of the metabolic status of the cell but are also a hub for cell death and innate immunity signaling pathways [47], we wanted to determine whether F1 impairs the response of innate immune cells to MVA-C infection. We analyzed by real-time PCR and ELISA the expression of type I IFNs (IFN-α and IFN-β), pro-inflammatory cytokines (IL-1β, TNF, IL-6, IL-12p40) and chemokines (MIP-1α) by murine bone marrow-derived macrophages (BMDMs) and dendritic cells (BMDCs) mock-infected or infected with MVA-C or MVA-C-ΔF1L (Figure 4). Compared to MVA-C, MVA-C-ΔF1L markedly up-regulated type I IFN, cytokine and chemokine expression in both BMDMs and BMDCs. These differences in apoptosis induction and cytokine/chemokine expression may have an impact on the immunogenicity of the vector. Thus, we next analyzed the immune response elicited in mice by MVA-C-ΔF1L deletion mutant.

### Deletion of the Viral Gene *F1L* in MVA-C Induces High, Broad and Polyfunctional HIV-1-specific T Cell Adaptive Immune Responses

**Figure 5:**
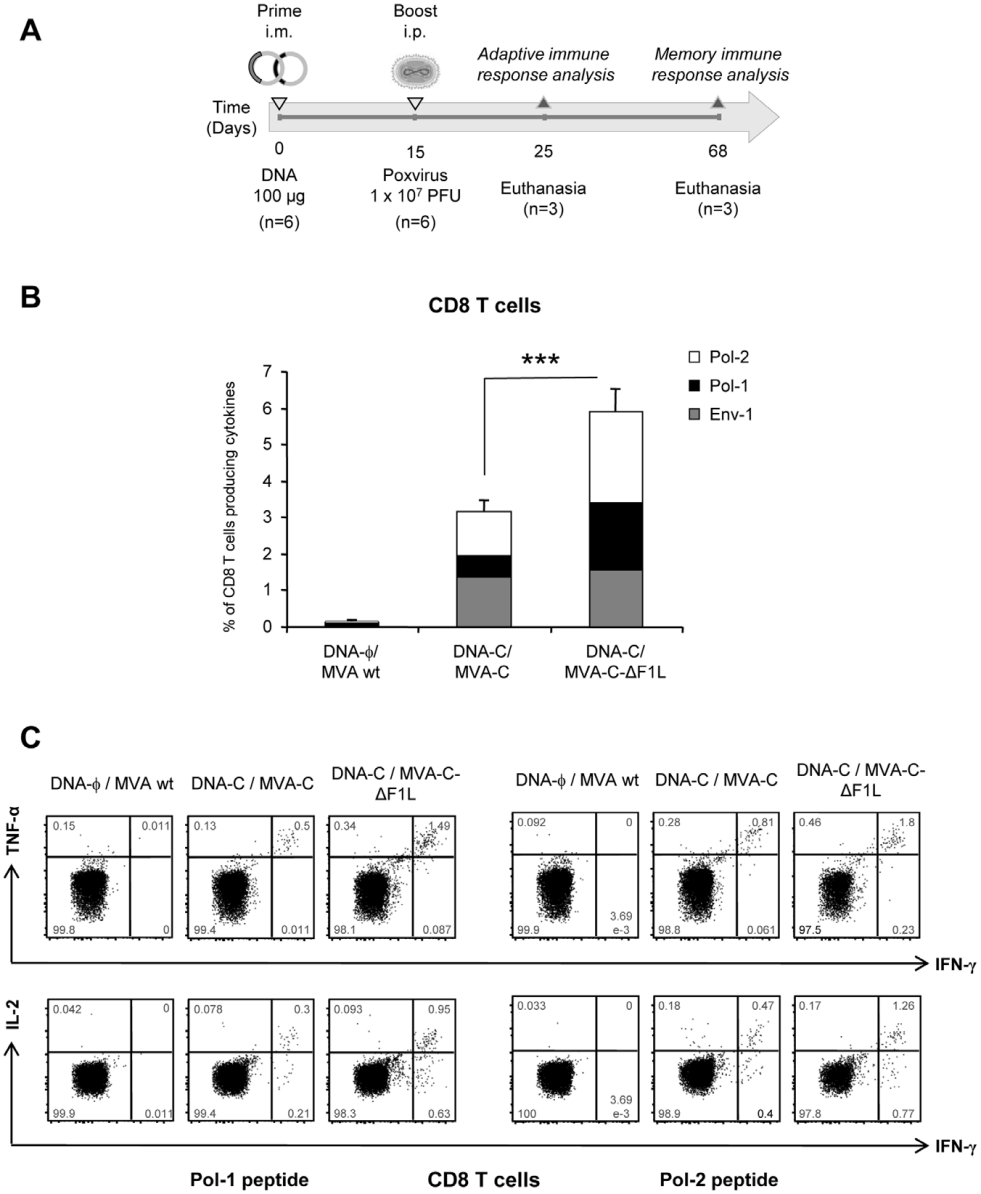
Adaptive HIV-1-specific T cell immune response elicited by *F1L* deletion mutant. (A) Schematic diagram showing the vaccination schedule followed in the study and the immunogenicity endpoints. (B) Magnitude of the vaccine-specific CD8 T cell response. The HIV-1-specific CD8 T cells were measured 10 days after the last immunization by ICS assay following stimulation with the different HIV-1 peptides in 3 mice of each group (n = 3). The total value in each group represents the sum of the percentages of CD8^+^ T cells secreting IFN-γ and/or IL-2 and/or TNF-α against Env-1+Pol-1+Pol-2 peptides. All data are background-subtracted. (C) Flow cytometry profiles of vaccine-induced CD8 T cell responses against Pol-1 or Pol-2 peptides. *** *p*<0.001. *p* value indicates significantly higher response compared to DNA-C/MVA-C immunization group.

To assay *in vivo* the effect of *F1L* deletion on the cellular immunogenicity against HIV-1 antigens, we analyzed the HIV-1-specific immune responses elicited by MVA-C-ΔF1L in mice using a DNA prime/Poxvirus boost approach since it has been extensively reported that this heterologous immunization protocol is more immunogenic than either component alone to activate T cell responses to HIV-1 antigens [44], [48], [49]. BALB/c mice, 6 in each group, were immunized according to the schedule shown in Figure 5A. Animals primed with sham DNA (DNA-φ) and boosted with the non-recombinant MVA wt were used as control group. Ten days after the boost (day 25), the adaptive T cell immune responses in 3 mice of each group were measured by polychromatic ICS assay after the stimulation of splenocytes with HIV-1 Env-1, Pol-1 or Pol-2 peptides. These 3 peptides were selected for the ICS analysis because they have been finely mapped and described as the most immunogenic MHC class I-restricted CTL peptides in the BALB/c model against the same clade C construct [50]. As shown in Figure 5B, the magnitudes of the total HIV-1-specific CD8 T cell response, determined as the sum of the individual responses obtained for Env-1, Pol-1 and Pol-2 peptides, were significantly higher in the groups immunized with MVA-C or MVA-C-ΔF1L than that obtained in the control group DNA-φ/MVA wt (*p*<0.001). Furthermore, the magnitude of the HIV-1-specific CD8 T cell response in the group immunized with MVA-C-ΔF1L was significantly higher than that obtained in the group DNA-C/MVA-C (*p*<0.001). This enhancement in the magnitude of the CD8^+^ T cell response observed in the animals immunized with MVA-C-ΔF1L was clearly directed against the Pol protein since the anti-Env response was not significantly affected. Representative profiles of Pol-induced CD8 T cell responses were shown in Figure 5C. Similar findings were observed in two independent experiments.

**Figure 6:**
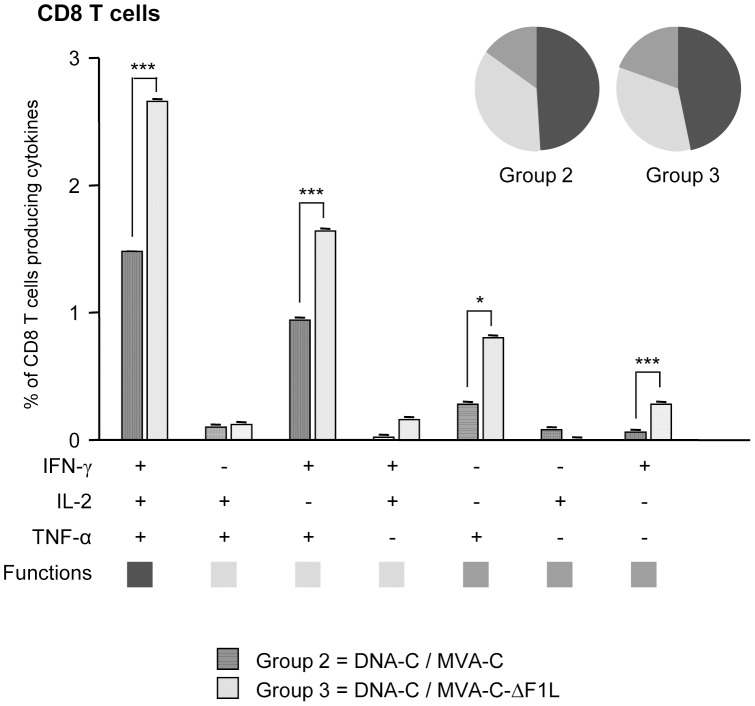
Functional profile of the adaptive HIV-1-specific CD8 T cell response in the different immunization groups. All the possible combinations of the responses are shown on the *x* axis, whereas the percentages of the functionally distinct cell populations within the total CD8 T cell population are shown on the *y* axis. Responses are grouped and color-coded on the basis of the number of functions. The non-specific responses obtained in the control group DNA-φ/MVA wt were subtracted in all populations. * *p*<0.05; *** *p*<0.001. *p* values indicate significantly higher responses compared to DNA-C/MVA-C immunization group.

The quality of a T cell immune response can be characterized in part by the pattern of cytokine secretion. On the basis of the analysis of IFN-γ, IL-2 and TNF-α production, seven different HIV-1-specific CD8 T cell populations were identified (Figure 6). The percentages of cells producing cytokines obtained in the DNA-φ/MVA wt control populations were subtracted in the DNA-C/MVA-C or DNA-C/MVA-C-ΔF1L immunization groups in order to remove the non-specific responses detected as background. Vaccine-induced CD8 T cell responses were highly polyfunctional in both immunization groups, with more than 75% of CD8^+^ T cells exhibiting two or three functions. CD8^+^ T cells producing IFN-γ+IL-2+TNF-α, IFN-γ+TNF-α and only TNF-α or IFN-γ were the most representative populations induced by the parental MVA-C and the deletion mutant MVA-C-ΔF1L, but the absolute frequencies of each population were significantly higher in the group boosted with MVA-C-ΔF1L compared with the parental MVA-C (Figure 6).

Since CD8 anti-Gag responses have been associated with better control of HIV/AIDS disease in individuals with chronic HIV-1 infection [51], we decided to determine the magnitude of the response triggered by Gag antigen by ELISPOT assay. Splenocytes isolated from immunized animals were stimulated with the HIV-1 peptide pools Gag-1, Gag-2 and GPN-1, which spanned the entire Gag and part of Pol antigens included in the immunogens. As shown in Figure S2, the magnitude of the total Gag-specific T cell response, determined as the sum of the individual responses obtained for the different peptide pools, was significantly higher in the group immunized with MVA-C-ΔF1L than in the group immunized with DNA-C/MVA-C (*p*<0.001).

Overall, these results indicate that deletion of *F1L* gene from MVA-C genome improved the magnitude of the HIV-1-specific adaptive CD8 T cell immune response and maintained the polyfunctional profile observed with the parental MVA-C. Since the contribution of the DNA prime is the same for MVA-C or MVA-C-ΔF1L immunization groups, the differences observed should be attributed to the *F1L* deletion.

### Deletion of the Viral Gene *F1L* Impacts on the CD8 T Cell Memory Phase of the Immune Response

**Figure 7:**
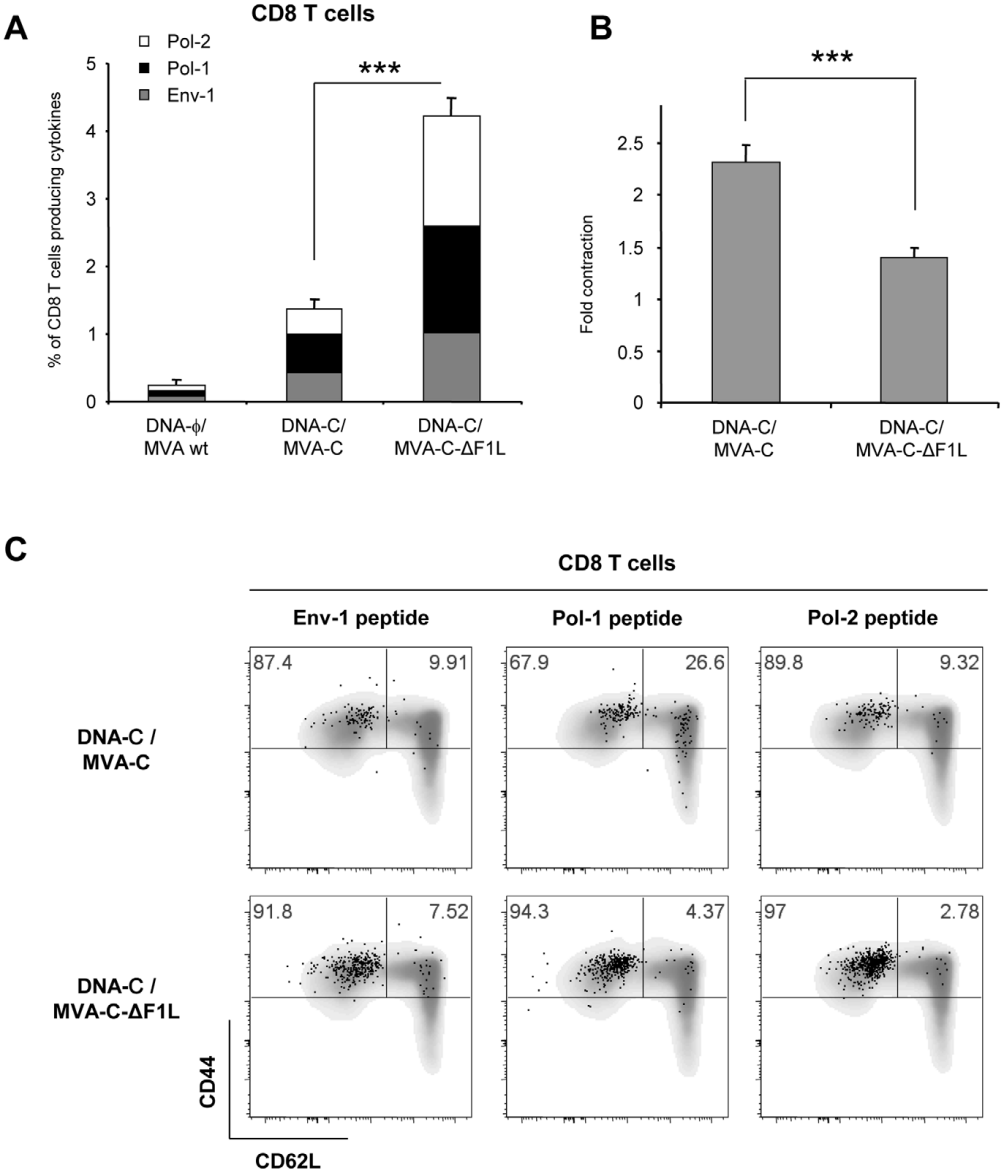
Memory response to HIV-1 peptides elicited by *F1L* deletion mutant. (A) Magnitude of vaccine-specific CD8 T cells. The HIV-1-specific CD8 T cells were measured 53 days after the last immunization by ICS assay following stimulation with the different HIV-1 peptides in the 3 mice left in each group (n = 3). The values represent the sum of the percentages of positive T cells secreting IFN-γ and/or IL-2 and/or TNF-α against Env-1+Pol-1+Pol-2 peptides. All data are background-subtracted. (B) Fold contraction of the frequency of HIV-1-specific CD8 T cells following boost. Fold contraction was calculated as a ratio of HIV-1-positive T cells at days 10 and 53 post boost. (C) Phenotypic profile of memory HIV-1-specific CD8 T cells. Representative FACS plots showing the percentage of Env-1, Pol-1 or Pol-2-specific CD8 T cells with central memory (TCM, CD44^high^CD62L^+^) or effector memory (TEM, CD44^high^CD62L^−^) phenotype. *** *p*<0.001. *p* values indicate significantly higher response (A) or lower fold contraction (B) compared to DNA-C/MVA-C immunization group.

Phenotypic analysis of memory vaccine-induced T cell response was performed 53 days after the last immunization by polychromatic ICS assay in the 3 mice left in each group (n = 3). Splenocytes from immunized mice were stimulated with the HIV-1 peptides Env-1, Pol-1 or Pol-2 for 6 hours and stained with specific antibodies to identify CD8 T cell lineage, responding cells (IFN-γ, IL-2 and TNF-α) as well as memory stages (CD44 and CD62L). As shown in Figure 7A, the magnitudes of the memory HIV-1-specific CD8 T cell response, determined as the sum of the individual responses obtained for Env-1, Pol-1 or Pol-2 peptides, were significantly higher in the groups boosted with the parental (MVA-C) or the deletion mutant (MVA-C-ΔF1L) than in the control group DNA-φ/MVA wt (*p*<0.001). Additionally, the magnitude of the memory HIV-1-specific CD8 T cell response in the group immunized with MVA-C-ΔF1L was significantly higher than that obtained in the group DNA-C/MVA-C (*p*<0.001). This enhancement in the magnitude of the CD8 T cell response observed in the animals immunized with MVA-C-ΔF1L was directed against both Env and Pol HIV-1 proteins but with preference towards Pol since individual responses against Env-1, Pol-1 or Pol-2 peptides were 2, 3 or 6-fold higher than in mice immunized with MVA-C, respectively.

Contraction of the CD8^+^ T cell pool, determined as the ratio of total HIV-1 positive cells at days 10 and 53 post-boost, was significantly reduced (*p*<0.001) in the group of mice inoculated with the deletion mutant MVA-C-ΔF1L in comparison with animals immunized with the parental MVA-C (Figure 7B).

We also determined the phenotype of the memory response by measuring the expression of CD62L and CD44 in the HIV-1-specific CD8 T cells. CD62L is a key marker that discriminates the effector and central memory T cell subpopulations in combination with CD44, which is expressed at high levels in all effector and central memory but not in naïve T cells [52]. Thus, the effector memory T cells (TEM) have a CD44^high^CD62L^−^ phenotype whereas the central memory T cells (TCM) are CD44^high^CD62L^+^. For CD8 T cells, the HIV-1-specific memory responses elicited by MVA-C and MVA-C-ΔF1L were predominantly TEM but some differences were observed between both groups. After MVA-C boost, 67.9% and 89.8% of the CD8^+^ T cells against Pol-1 and Pol-2 peptides respectively were TEM, whereas the deletion of *F1L* increased these populations up to 90% (94.3% and 97%) (Figure 7C). Overall, these results indicated that single deletion of the viral gene *F1L* impacts on the CD8 T cell memory phase of the immune response enhancing the magnitude of the response, reducing the contraction phase and changing the memory differentiation pattern.

Since cells infected with MVA-C release monomeric gp120 [44] and it is generally accepted that both cellular and humoral arms of the immune system are necessary to control HIV infection [53], we quantified by ELISA the Env-specific IgG binding antibodies in the serum of immunized animals during the memory phase of the immune response. We observed that in the group boosted with the *F1L* deletion mutant, the levels of anti-gp120 antibodies were higher than those obtained in animals immunized with the parental MVA-C, although the difference was not statistically significant (data not shown).

## Discussion

A number of unique features make poxvirus recombinants good candidates as vaccine vectors and therefore members of the *Poxviridae* family are being widely used in the prevention and treatment of emergent infectious diseases and cancer, with an increasing interest focused on the highly attenuated ALVAC, MVA and NYVAC strains [42], [43]. Although these attenuated VACV strains exert good safety and immunogenicity profiles, the development of more efficient candidate vectors that enhance the magnitude, breadth, polyfunctionality and durability of the immune response against heterologous antigens is needed, especially after the 31.2% of protection against HIV infection obtained in the recent phase III clinical trial (RV144) in Thailand using a combination of a recombinant ALVAC and the protein gp120 [1]. One strategy to afford this objective consists in the removal of specific viral immunomodulatory genes involved in the inhibition of the host anti-viral response. This methodology has been successfully used for MVA and NYVAC vectors [3], [54], [55], [56], [57], [58], [59], [60], [61]. Here, we have evaluated another different approach based on the removal of a viral inhibitor of apoptosis with the aim to optimize poxvirus-based vectors.

It has been previously reported that DCs that have phagocytosed apoptotic infected cells can cross-present viral or tumor antigens to cytotoxic T cells and induce a cytotoxic response [27], [62]. Therefore, we have examined the effect of the deletion of the viral anti-apoptotic gene *F1L* on the immunogenicity of the HIV/AIDS vaccine candidate MVA-C in a BALB/c mouse model. The MVA-C recombinant, expressing the HIV-1 gp120 and Gag-Pol-Nef proteins from clade C, has been previously characterized [44], [63] and its immunogenic potential has been reported in the murine model [44].

In the present study, we have shown that viral gene *F1L* does not affect virus replication nor HIV-1 antigen expression in cultured cells and its deletion in MVA-C increases the percentage of early apoptotic cells in murine and human cell lines and in bone marrow-derived dendritic cells and enhances the expression of type I IFN and pro-inflammatory cytokine genes in murine macrophages and dendritic cells. Using a DNA-C prime/MVA-C boost protocol in BALB/c mice, we have observed that deletion of *F1L* from MVA-C genome, improves the magnitude of the HIV-1-specific CD8 T cell adaptive and memory immune responses maintaining a highly polyfunctional profile. This enhancement in the HIV-1-specific CD8 T cell responses induced in animals boosted with the MVA-C-ΔF1L deletion mutant might be attributed to the increased cross-presentation of viral antigens by DCs that have engulfed apoptotic MVA-C-ΔF1L-infected cells.

Antigens derived from viral infections can be recognized by cytotoxic T cells (CTLs) if such viral antigens are presented by DCs, the most potent antigen-presenting cells (APCs) and the only cell type capable of activating naïve T cells [64], [65], [66]. DCs are specialized to process and present in a major histocompatibility complex (MHC) class I-restricted manner viral antigens synthesized endogenously within the infected DC itself (direct presentation) or exogenously from acquired antigens produced by other infected cells (cross-presentation) [67]. Although MVA has the ability to infect DCs which are capable to express and present viral and heterologous antigens to CTLs [68], the abortive viral cell cycle at early stages, the apoptosis induced in DCs after MVA infection (even more accelerated in the case of DCs infected with MVA-C-ΔF1L) and the host cell protein synthesis shutdown, point to the conclusion that cross-presentation could be the dominant pathway for the priming of CD8^+^ T cells in response to MVA infection [69], [70]. In this context, cross-presentation of tumor and viral antigens expressed by different poxviruses, including MVA, has been extensively documented [41], [71], [72].

According to the poxvirus inoculation route used in this immunization schedule (i.p.), the effect of *F1L* gene deletion on immunogenicity against HIV-1 antigens, should be explained by the impact of enhanced apoptosis induction in the cell types present in the peritoneal cavity, which are mainly B cells, macrophages and granulocytes and, to a lesser extent, T cells [73]. It has been reported that MVA preferentially infects DCs, monocytes/macrophages and B cells in mice and humans [74], [75], [76], [77] and induces apoptosis in these cell types [40], [77]. In this context, cell death induction by MVA may be desirable for antigen uptake and cross-presentation by uninfected DCs and this cross-presentation could be further enhanced in animals immunized with MVA-C-ΔF1L since *F1L* deletion mutant triggers apoptosis earlier than MVA-C. In this context, it has been reported that human DCs exposed to MVA initiate apoptosis at least 1 day earlier that DCs exposed to VACV [70] and this early apoptosis induction may explain, at least in part, why the immunogenicity of MVA is equal to or greater than that of replication-competent strains of VACV [78], [79], [80], [81].

On the other hand, MVA has been shown to induce a robust innate immune response characterized by the production of pro-inflammatory cytokines (TNF, IL-1β, IL-6, IL-12p40), chemokines (IP-10/CXCL10, RANTES/CCL5, MCP-5/CCL12, MIP-2/CXCL2) and type I interferon (IFN-β) in murine peritoneal cells and peritoneal lavage fluid [82]. In the present study, we have demonstrated that MVA-C is also able to induce the secretion of type I IFNs, chemokines (MIP-1α) and pro-inflammatory cytokines (TNF, IL-1β, IL-6, IL-12p40) in murine bone marrow-derived macrophages and dendritic cells and that deletion of *F1L* gene enhanced this type I IFN and cytokine/chemokine production. MVA infection has also been reported to induce robust production of chemokines in human macrophages/monocytes [82], [83] and this observation correlated with the results obtained in our study in which MVA-C-ΔF1L infection of human THP-1 macrophages markedly up-regulated IFN-β as well as IL-8 and IP-10 chemokines production (data not shown). In the context of an intraperitoneal immunization with MVA-C-ΔF1L deletion mutant, the increased secretion of pro-inflammatory cytokines and chemokines by infected macrophages could produce an enhanced recruitment of immature DCs and lymphocytes, thus generating an appropriate environment for the cross-presentation of vaccine-encoded antigens. Immature DCs that have engulfed apoptotic MVA-C-ΔF1L-infected cells can also migrate to the lymph nodes, maturing in route, and activate antigen-specific CD8 T cells. The recruitment of immature DCs to the site of infection may also occur when the number of apoptotic cells overcomes the scavenger potential of macrophages, which may occur in mice immunized with MVA-C-ΔF1L. Therefore, immunogenicity against the dying cells might also be influenced by the relative amounts of cells undergoing apoptosis at a given time.

Taking together all the above considerations, the observation that the magnitude of the HIV-1-specific CD8 T cell adaptive response in the group immunized with MVA-C-ΔF1L was significantly higher than that obtained in the group DNA-C/MVA-C (*p*<0.001) could be attributed to an enhanced cross-presentation of HIV-1 antigens by *F1L* gene deletion-induced apoptosis of infected cells together with the increased pro-inflammatory environment generated. The observation that this enhancement in the magnitude of the CD8 T cell response was clearly directed against the Pol protein is in concordance with the intracellular nature of the Gag-Pol-Nef immunogen. Immunization with MVA-C-ΔF1L does not decrease the polyfunctional profile of the HIV-1-specific CD8 T cells compared to that obtained with MVA-C inoculation, since vaccine-induced CD8 T cell responses were highly polyfunctional in both immunization groups, with more than 75% of CD8^+^ T cells exhibiting two or three functions.

During the memory phase of the immune response, the magnitude of the HIV-1-specific CD8 T cell response in the group immunized with MVA-C-ΔF1L was also significantly higher than that obtained in the group DNA-C/MVA-C, again with a clear preference towards Pol antigen (*p*<0.001). This increase in the magnitude of the memory response could be a consequence of the enhanced HIV-1-specific CD8 T cell response elicited during the adaptive phase of the immune response. Additionally, the CD8^+^ T cells activated by *F1L* deletion mutant during the memory phase have enhanced differentiation to an effector memory phenotype (TEM) compared with the CD8^+^ T cells activated by the parental MVA-C, particularly against Pol-1 peptide. This is of particular relevance since an important role of the effector memory T cells on the early control of highly pathogenic SIV has been reported [84], [85].

The effect on the HIV-specific immune responses elicited by attenuated strains of VACV expressing different HIV-1 antigens and lacking several immunomodulatory genes has been previously reported in the context of DNA prime/Poxvirus boost immunization protocols. A recombinant MVA expressing Env and Gag-Pol-Nef of HIV-1 from clade B (MVA-B) with *A41L* and *B16R* genes deleted, has been shown to significantly enhance the magnitude of the HIV-1-specific adaptive and memory T cell responses [57]. The total magnitudes obtained with the double deletion mutant were about 13% (adaptive phase) and 22% (memory phase) compared with the 5% and 11% obtained in animals immunized with the combination DNA-B/MVA-B, respectively [57]. These enhanced HIV-1-specific immune responses were mostly mediated by GPN-specific CD8^+^ T cells while T cells induced by the parental MVA-B were mainly Env-specific CD8^+^ T cells [57]. An enhancement in the HIV-1-specific memory T cell response has also been reported in the case of an MVA-B recombinant lacking *C6L* gene [3], with the HIV-1-specific memory T cell immune response (11% vs. 3%) mainly mediated by GPN-specific CD8^+^ T cells [3]. This immunodominance towards CD8^+^ GPN-specific T cell immune response is in concordance with the results obtained with the *F1L* deletion mutant in which 6% vs. 3% (adaptive phase) and 4% vs. 1% (memory phase) of HIV-specific CD8^+^ T cells were mainly directed against Pol antigen. The lower magnitudes obtained with the *F1L* deletion mutant could be attributed to the more restricted stimulation used (Env-1, Pol-1 or Pol-2 peptides compared with a wide panel of Env, Gag and GPN peptide pools). This modulation of the HIV-1-specific immune responses by the removal of immunomodulatory genes has also been reported in the case of DNA Prime/NYVAC boost immunization schedules. NYVAC-C (expressing Env and Gag-Pol-Nef of HIV-1 from clade C) deletion mutants lacking *B19R* and/or *B8R* genes, encoding inhibitors of type I and type II IFNs, respectively, have also been shown to improve the magnitude of HIV-1-specific CD8^+^ T cell adaptive immune responses and to impact their memory phase [61]. HIV-1-specific CD8^+^ T cell responses were higher in the groups boosted with the NYVAC-C deletion mutants (2.5%–4%) than in the group boosted with the parental NYVAC-C (1.7%) and this enhancement is clearly directed against the Env pool [61]. However, during the memory phase of the immune response, the patterns of CD8^+^ T cells were different between the vectors with NYVAC-C, NYVAC-C-ΔB19R and NYVAC-C-ΔB8R/ΔB19R inducing a higher percentage of GPN-specific CD8^+^ T cells (NYVAC-C-ΔB8R/ΔB19R > NYVAC-C-ΔB19R) and NYVAC-C-ΔB8R eliciting preferentially Env-specific CD8^+^ T cells [61]. The above observations show that the deletion of immunomodulatory genes that antagonize host-specific immune responses enhances the HIV-specific immune responses and suggest that multiple combinations of such deletions could be a strategy for the generation of optimized poxvirus-based HIV vaccine candidates.

In summary, the results of the present work indicate that single deletion of the viral gene *F1L* improves the magnitude of the HIV-1-specific CD8 T cell adaptive immune response and impacts on the CD8 T cell memory phase increasing the magnitude of the response, reducing the contraction phase and changing the memory differentiation pattern by possibly enhancing the cross-presentation of HIV-1 antigens by DCs mediated by apoptosis induction.

These findings may have implications for vaccine design since *F1L* deletion from MVA vaccines may constitute a mechanism to deliver heterologous antigens in a manner that will promote cross-presentation of such antigens and hence stimulating strong immunity. The observation that F1 is currently the only VACV Bcl-2-like protein with anti-apoptotic activity, since the other proposed Bcl-2-like protein N1 has been reported to inhibit the innate immunity signaling pathways that activate IRF-3 and NF-κB transcription factors but not to inhibit apoptosis [37], [86], [87], suggests that F1 can be considered both as an anti-apoptotic protein and as an immune modulator. In summary, these findings reveal the immunomodulatory role of F1 and that deletion of the viral anti-apoptotic *F1L* gene could be a valid strategy for the optimization of MVA as vaccine vector.

## Materials and Methods

### Ethics Statement

The animal studies were approved by the Ethical Committee of Animal Experimentation (CEEA-CNB) of Centro Nacional de Biotecnologia (CNB-CSIC, Madrid, Spain) in accordance with national and international guidelines and with the Royal Decree (RD 1201/2005) (Permit numbers: 152/07 and 080030) and by the Office Vétérinaire du Canton de Vaud, Switzerland (authorizations n° 876.7 and 877.7).

### Cells and Viruses

Primary chicken embryo fibroblasts (CEF), DF-1 cells (a spontaneously immortalized chicken embryo fibroblast cell line, ATCC, Cat. No. CRL-12203), 3T3 cells (a mouse fibroblast-derived cell line, ATCC, Cat. No. CCL-92) and HeLa cells (a human epithelial cervix adenocarcinoma, ATCC, Cat. No. CCL-2) were grown in Dulbeccós modified Eaglés medium (DMEM) supplemented with 10% fetal calf serum (FCS). Murine bone marrow cells were cultured for 7 days in IMDM (Invitrogen) supplemented with 10% FCS containing 50 µM 2-mercaptoethanol and monocyte-colony stimulating factor or granulocyte-monocyte colony stimulating factor to obtain bone marrow-derived macrophages (BMDMs) or dendritic cells (BMDCs), respectively. All media were supplemented with 100 IU/ml of penicillin and 100 µg/ml of streptomycin. Cells were maintained in a humidified air 5% CO_2_ atmosphere at 37°C (or 39°C for DF-1 cell line). Virus infections were performed with 2% FCS for all cell types. The poxvirus strains used in this work include modified vaccinia virus Ankara (MVA) obtained from the Ankara strain after 586 serial passages in CEF cells (derived from clone F6 at passage 585, kindly provided by G. Sutter, Germany) and MVA-C expressing gp120 as a cell released product and Gag-Pol-Nef as an intracellular polyprotein from the clade C CN54 HIV-1 isolate [44], used as parental virus for the generation of the *F1L* deletion mutant. All viruses were grown in CEF cells, similarly purified through two 36% (w/v) sucrose cushions and titrated by immunostaining plaque assay as previously described [81]. The titration of the different viruses was performed at least three times.

### Construction of Plasmid Transfer Vector pGem-RG-F1L

The plasmid transfer vector pGem-RG-F1L, used for the construction of recombinant virus MVA-C-ΔF1L in which *F1L* ORF has been replaced for a GFP expression cassette, was obtained by the sequential cloning of four DNA fragments containing dsRed2 and rsGFP genes and *F1L* recombination flanking sequences into the plasmid pGem-7Zf(-) (Promega). The dsRed2 gene under the control of the synthetic early/late promoter (783 bp) was obtained by digestion of plasmid pG-dsRed2 (encoding dsRed2 and rsGFP genes) with Xba I and Eco RI and inserted into the Xba I/Eco RI-digested pGem-7Zf(-) to generate pGem-Red (3755 bp). The rsGFP gene under the control of the synthetic early/late promoter was amplified by PCR from plasmid pG-dsRed2 with oligonucleotides GFP-C (5′-GTTGGATCGATGAGAAAAATTG-3′) (Cla I site underlined) and GFP-B (5′-CTATAGGATCCTCAAGCTATGC-3′) (Bam HI site underlined) (828 bp), digested with Cla I and Bam HI and inserted into plasmid pGem-Red previously digested with Cla I and Bam HI to obtain pGem-Red-GFP (4555 bp). MVA genome was used as template to amplify the left flank of *F1L* gene (351 bp) with oligonucleotides fiF1L-BF (5′-CATCGAGGATCCACTATTGTTTAT-3′) (Bam HI site underlined) and fiF1L-BR (5′-TTATAGGATCCCTCCAGGAGAAAG-3′) (Bam HI site underlined). This left flank was digested with Bam HI and cloned into plasmid pGem-Red-GFP previously digested with the same restriction enzyme to generate pGem-RG-fiF1L (4889 bp). The right flank of *F1L* gene (382 bp) was amplified by PCR from MVA genome with oligonucleotides fdF1L-S (5′-CCAGTCCCGGGAGACTGTACAA-3′) (Sma I site underlined) and fdF1L-C (5′-GATAATCGATTTTTTTTTAACACG-3′) (Cla I site underlined), digested with Sma I and Cla I and inserted into the Sma I/Cla I-digested pGem-RG-fiF1L. The resulting plasmid pGem-RG-F1L (5244 bp; Figure 1A) was confirmed by DNA sequence analysis and directs the insertion of GFP gene into *F1L* locus of MVA-C.

### Construction of MVA-C-ΔF1L Deletion Mutant

MVA-C-ΔF1L was constructed using dsRed2 and rsGFP proteins as fluorescent markers. 3×10^6^ DF-1 cells were infected with 0.05 PFU/cell of MVA-C and transfected 1 hour later with 8 µg DNA of plasmid pGem-RG-F1L using Lipofectamine 2000 (Invitrogen) according to the manufactureŕs recommendations. After 48 hours post-infection, the cells were harvested, lysed by freeze-thaw cycling, sonicated and used for recombinant virus screening. MVA-C-ΔF1L was selected from progeny virus by consecutive rounds of plaque purification in DF-1 cells during which plaques were screened for Red2/GFP fluorescence. In the first three passages viruses from selected plaques expressed both fluorescent proteins while in the last three passages (six passages in total) viral progeny from selected plaques expressed only GFP due to the loss of dsRed2 marker.

### PCR Analysis of MVA-C-ΔF1L Deletion Mutant

To test the identity and purity of the recombinant virus MVA-C-ΔF1L, viral DNA was extracted from DF-1 cells infected at 5 PFU/cell with MVA wt or MVA-C-ΔF1L. Cell membranes were disrupted using sodium dodecyl sulphate (SDS) followed by proteinase K treatment (0.2 mg/ml proteinase K in 50 mM Tris-HCl pH 8, 100 mM EDTA pH 8, 100 mM NaCl and 1% SDS for 1 hour at 55°C) and phenol extraction of viral DNA. Primers fdF1L-S and fiF1L-BR spanning *F1L* flanking regions were used for PCR analysis of *F1L* locus. The amplification reactions were carried out with Platinum Taq DNA polymerase (Invitrogen) according to the manufactureŕs recommendations.

### Analysis of Virus Growth

To determine virus growth profiles, monolayers of CEF cells grown in 12-well plates were infected in duplicate at 0.01 PFU/cell with MVA-C or MVA-C-ΔF1L deletion mutant. Following virus adsorption for 60 min at 37°C, the inoculum was removed. The infected cells were washed once with DMEM without serum and incubated with fresh DMEM containing 2% FCS at 37°C in a 5% CO_2_ atmosphere. At different times post-infection (0, 24, 48 and 72 hours), cells were harvested by scraping (lysates at 5×10^5^ cells/ml), freeze-thawed three times and briefly sonicated. Virus titers in cell lysates were determined by immunostaining assay in DF-1 cells using rabbit polyclonal anti-vaccinia virus strain WR (Centro Nacional de Biotecnología; diluted 1∶1000), followed by anti-rabbit-HRP (Sigma; diluted 1∶1000).

### Genetic Stability of MVA-C-ΔF1L Deletion Mutant by HIV-1 Antigens Expression Analysis

To analyze the stability of HIV-1 antigens expressed by *F1L* deletion mutant, monolayers of DF-1 cells grown in 6-well plates were infected with serial dilutions of purified MVA-C-ΔF1L. After 1 hour of virus adsorption, the virus inoculum was removed and cells overlayed with agar. At 48 hours post-infection, cells were stained with 0.01% neutral red (Sigma) and 15 hours later 30 individual plaques were picked up, resuspended in 0.5 ml of DMEM, freeze-thawed three times and briefly sonicated. 0.2 ml of each plaque was used for infection of DF-1 cells in 24-well plates. At 72 hours post-infection, cells were lysed in Laemmli buffer containing β-mercaptoethanol, cells extracts fractionated by 8% SDS-PAGE and analyzed by Western-blot using rabbit polyclonal anti-gp120 antibody (Centro Nacional de Biotecnología; diluted 1∶3000) or polyclonal anti-gag p24 serum (ARP 432, NIBSC, Centralised Facility for AIDS reagent, UK; diluted 1∶1000) followed by anti-rabbit-HRP (Sigma; diluted 1∶5000) to evaluate the expression of gp120 and GPN proteins, respectively. The immunocomplexes were detected by enhanced chemiluminescence (ECL, GE Healthcare).

### Identification of GPN Mutations

Viral DNA was extracted by the method of SDS-Proteinase K-Phenol from DF-1 cells infected with different isolated plaques of MVA-C-ΔF1L and used as template to amplify specific regions of GPN polyprotein. The amplification reactions were carried out with Platinum Pfx DNA polymerase (Invitrogen) according to the manufactureŕs recommendations. Different PCR products were sequenced by Secugen, S.L. Primers used for PCR amplification and sequencing are depicted in Figure 2B.

### Measurement of Apoptosis by PARP Cleavage

The cleavage of poly ADP-ribose polymerase (PARP) was analyzed by Western-blot at 16 hours post-infection in extracts from HeLa cells grown in 12-well plates mock-infected or infected with MVA wt, MVA-C or MVA-C-ΔF1L at 5 PFU/cell. Rabbit polyclonal anti-human PARP (diluted 1∶500) was supplied by Cell Signaling and mouse monoclonal antibody against β-actin (diluted 1∶2000), anti-rabbit-HRP (diluted 1∶5000) and anti-mouse-HRP (diluted 1∶2000) were supplied by Sigma.

### Measurement of Apoptosis by Annexin V binding Assay

Annexin V binding assay was performed in combination with propidium iodide (PI) staining to monitor the integrity of the cell membrane. 5×10^5^ HeLa, 3T3 cells or murine DCs were infected with MVA wt, MVA-C or MVA-C-ΔF1L at 5 PFU/cell. Mock-infected cells and cells treated with staurosporine (0.5 µM; Sigma) were used as negative and positive controls, respectively. At 16 hours post-infection, floating and adhered cells were collected from the wells by pipetting and centrifuged at 3000 rpm for 5 min at 4°C. Cells were washed twice with cold phosphate buffered saline (PBS) and resuspended in 0.1 ml of binding buffer (10 mM Hepes pH 7.4, 140 mM NaCl, 5 mM CaCl_2_). Cells were stained with Annexin V-Biotin (Beckman Coulter) for 15 min at 4°C in the dark according to the manufactureŕs recommendations. After one wash with binding buffer, cells were incubated with Streptavidin-PE (Beckman Coulter, diluted 1∶100) for 20 min at room temperature. Finally, cells were washed once with binding buffer and resuspended in 0.5 ml binding buffer containing PI (1 µg/ml; Beckman Coulter). The percentage of apoptotic cells was determined by flow cytometry. 1×10^5^ cells were acquired using an LSRII flow cytometer (BD Biosciences). Analyses of the data were performed using the FlowJo software version 8.5.3 (Tree Star, Ashland, OR). Early apoptotic cells were defined as Annexin V positive and PI negative.

### RNA Analysis by Quantitative Real-time Polymerase Chain Reaction

Total RNA was isolated using the RNeasy kit (Qiagen) from murine BMDMs or BMDCs mock-infected or infected at 1 or 5 PFU/cell with MVA-C or MVA-C-ΔF1L for 6 hours. Reverse transcription of 100 ng to 500 ng of RNA was performed using the ImProm II RT System kit (Promega). Quantitative PCR was performed with a 7500 Fast Real-Time PCR System (Applied Biosystems) using the Power SYBR Green PCR Master Mix (Applied Biosystems). Expression levels of *Ifna, Ifnb, Ccl3 (Mip1a,) Il1b, Il6, Il12b, Tnf* and *Hprt* genes were analyzed by real-time PCR using specific oligonucleotides (sequences will be provided upon request). Gene specific expression was expressed relative to the expression of *Hprt* in arbitrary units (A.U.). All samples were tested in duplicate and two different experiments were performed.

### Measurement of Cytokine Production

A screening of mediators produced by murine BMDMs or BMDCs mock-infected or infected at 1 or 5 PFU/cell with MVA-C or MVA-C-ΔF1L for 24 hours was performed by ELISA. Mouse IFN-α, IFN-β (Biomedical Laboratories), MIP-1α, IL-1β, TNF, IL-6 and IL-12p40 (R&D systems) were quantified by ELISA. All samples were tested in duplicate and two different experiments were performed.

### DNA Vectors

The two DNA constructs expressing the HIV-1 _CN54_gp120 (pcDNA-_CN54_gp120) and HIV-1 _CN54_Gag-Pol-Nef (GPN) polyprotein (pcDNA-_CN54_GPN) have been previously reported [44]. Plasmids were purified using Maxi-prep purification kits (Qiagen) and diluted for injection in endotoxin-free PBS.

### Peptides

The HIV-1 peptides Env-1 (sequence: PADPNPQEM), Pol-1 (sequence: LVGPTPVNI) and Pol-2 (sequence: YYDPSKDLI) were previously described as H-2^d^-Restricted CTL epitopes [50] and were provided by the Proteomic service at the CNB-CSIC, Spain. The HIV-1 peptide pools Gag-1, Gag-2 and GPN-1 were provided by the EuroVacc Foundation and were previously described [44]. They spanned the entire HIV-1 Gag and part of Pol antigens included in the immunogens as consecutive 15-mers overlapping by 11 amino acids.

### Mouse Immunization Schedule

BALB/c mice (6–8 weeks old) were purchased from Harlan. For the heterologous DNA prime/MVA boost immunization protocol performed to assay the immunogenicity of the MVA-C-ΔF1L deletion mutant, groups of animals (n = 6) received 100 µg of DNA-C (50 µg of pcDNA-_CN54_gp120+50 µg of pcDNA-_CN54_GPN) or 100 µg of DNA-φ (100 µg of pcDNA) by intramuscular route (i.m.). Two weeks later, animals were immunized with 1×10^7^ PFU of MVA wt, MVA-C or MVA-C-ΔF1L by intraperitoneal route (i.p.). Mice immunized with sham DNA (DNA-φ) followed by MVA wt boost were used as control group. At 10 and 53 days after the last immunization, 3 mice in each group were sacrificed and spleens processed for Intracellular Cytokine Staining (ICS) assay to measure the adaptive and memory cellular immune responses against HIV-1 antigens, respectively. Two independent experiments have been performed for the different groups.

### Intracellular Cytokine Staining Assay (ICS)

The magnitude, polyfunctionality and phenotype of the HIV-1 specific T cell responses were analyzed by ICS. After an overnight rest, 4×10^6^ splenocytes (depleted of red blood cells) were seeded on 96-well plates and stimulated during 6 hours in complete RPMI 1640 media supplemented with 10% FCS containing 1 µl/ml GolgiPlug (BD Biosciences) and 20 µg/ml, 5 µg/ml or 10 µg/ml of the HIV-1 peptides Env-1, Pol-1 or Pol-2, respectively. At the end of the stimulation period, cells were washed, stained for the surface markers, fixed and permeabilized (Cytofix/Cytoperm Kit; BD Biosciences) and stained intracellularly using the appropriate fluorochromes. Dead cells were excluded using the violet LIVE/DEAD stain kit (Invitrogen). For functional analyses the following fluorochromes-conjugated antibodies were used: CD4-Alexa 700, CD8–FITC, IFN-γ-PE-Cy7, IL-2-APC, TNF-α-PE-Cy7 (all from BD Biosciences), CD3-FITC (Beckman Coulter), CD8-PerCP (BioLegend), IFN-γ-APC and IL-2-PE (both from eBioscience). In addition, for phenotypic analyses the following antibodies were used: CD62L-PE (SouthernBiotech) and CD44-SPRD (Beckman Coulter). Cells were acquired using an LSRII flow cytometer (BD Biosciences). Analyses of the data were performed using the FlowJo software version 8.5.3 (Tree Star, Ashland, OR). The number of lymphocyte-gated events ranged between 1×10^5^ and 1×10^6^. After gating, Boolean combinations of single functional gates were then created using FlowJo software to determine the frequency of each response based on all possible combinations of cytokine expression or all possible combinations of differentiation marker expression. Background responses detected in negative control samples were subtracted from those detected in stimulated samples for every specific functional combination.

### ELISPOT Assay

The Gag-specific adaptive cellular immune response was evaluated 10 days after the last immunization in a fresh IFN-γ enzyme-linked immunosorbent spot (ELISPOT) assay as previously described [88]. The HIV-1 peptide pools were resuspended in RPMI 1640 supplemented with 10% FCS and added to cells at a final concentration of 5 µg/ml.

### Data Analysis and Statistics

For the statistical analysis of ICS data, we used a novel approach that corrects measurements for the medium response (RPMI) and allows the calculation of confidence intervals and *p* values of hypothesis tests [57], [89]. Only antigen responses values significantly higher than the corresponding RPMI are represented and the background for the different cytokines in the unstimulated controls never exceeded 0.05%. The data analysis program, Simplified Presentation of Incredibly Complex Evaluations (SPICE, version 4.1.5, Mario Roederer, Vaccine Research Center, NIAID, NIH), was used to analyze and generate graphical representations of T cell responses detected by polychromatic flow cytometry. All values used for analyzing proportionate representation of responses are background-subtracted. For the analysis of Annexin V binding assay data, we used the same statistical procedure with minor modifications according to the specific experimental approach.

For the comparison of the ratio between adaptive and memory immune responses (contraction fold), we have developed a technique based on that previously reported [89]. Let us call 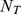 the total number of cells being analyzed, and 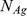 the number of cells responding to a given antigen. As in [89] we assume that the estimate of the proportion of cells responding to the antigen, 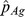, follows a Beta distribution with parameters 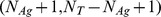. Let us call 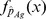
the probability density function associated to the proportion estimate. Analogously, we estimate the background response as the proportion of cells responding to the medium alone (RPMI), 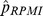 (being 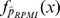 its corresponding probability density function). We define the corrected proportion as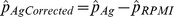. The probability density function of this new estimate is given by standard statistical rules, 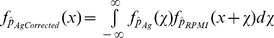. This corrected estimate of the proportion and its distribution are computed for the adaptive and memory responses. Then, we can compute the response contraction ratio as 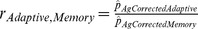, whose statistical distribution is given by 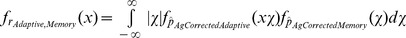. Finally, we compute the statistical distribution of the differences between the contraction ratio for different kind of virus strains. 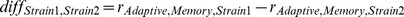. The probability density function of this new variable is given by 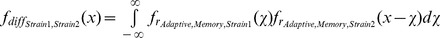. The *p* value of the hypothesis that Strain 1 has a ratio significantly larger than Strain 2 is given by
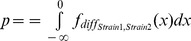
.

## Supporting Information

Figure S1 **CN54 clade C Gag-Pol-Nef sequence (4254 bp).** Cytosine-rich regions are depicted. GPN sequence contains 14 sequences of 5 cytosines (solid line), 1 region of 8 cytosines (shaded; cytosine insertion in plaque 19) and 1 region of 6 cytosines (dotted line; cytosine insertion in plaque 26).
(TIF)

Figure S2 **Adaptive Gag-specific T cell immune response elicited by** ***F1L*** **deletion mutant.** Magnitude of the total Gag-specific T cell response was measured 10 days after the last immunization by ELISPOT assay following stimulation with the different HIV-1 peptide pools in 3 mice of each group (n = 3). Number of IFN-γ-secreting cells are represented. *** *p*<0.001. *p* value indicates significantly higher response compared to DNA-C/MVA-C immunization group.
(TIF)
