# Supplementary figures and images for: Deletion of the Viral Anti-Apoptotic Gene F1L in the HIV/AIDS Vaccine Candidate MVA-C Enhances Immune Responses against HIV-1 Antigens
Source: PLoS One. 2012 Oct 31;7(10):e48524. doi: 10.1371/journal.pone.0048524 (PMC3485360; doi:10.1371/journal.pone.0048524)

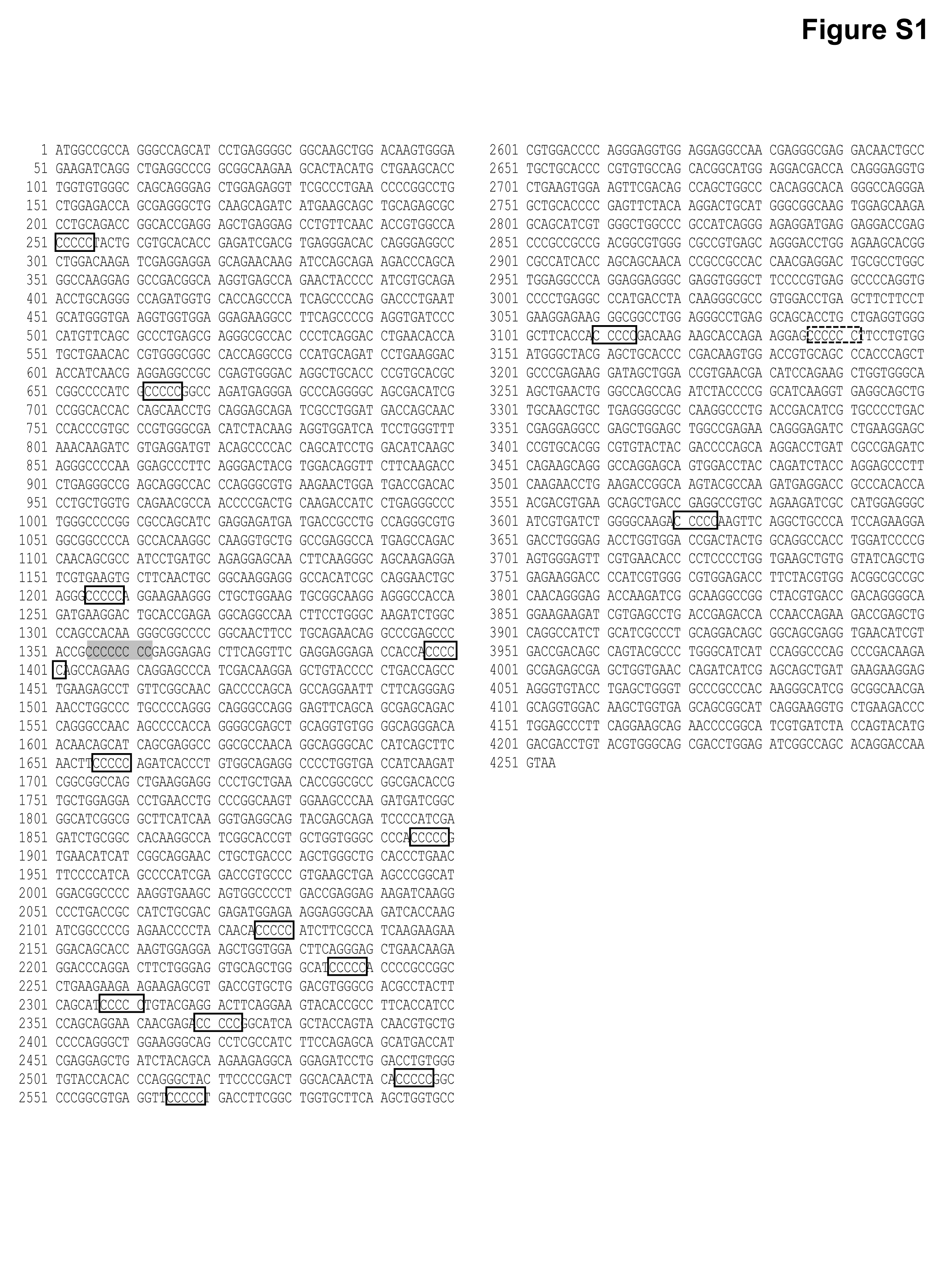

Supplement: Figure S1 — CN54 clade C Gag-Pol-Nef sequence (4254 bp). Cytosine-rich regions are depicted. GPN sequence contains 14 sequences of 5 cytosines (solid line), 1 region of 8 cytosines (shaded; cytosine insertion in plaque 19) and 1 region of 6 cytosines (dotted line; cytosine insertion in plaque 26). (TIF) [file pone.0048524.s001.tif]

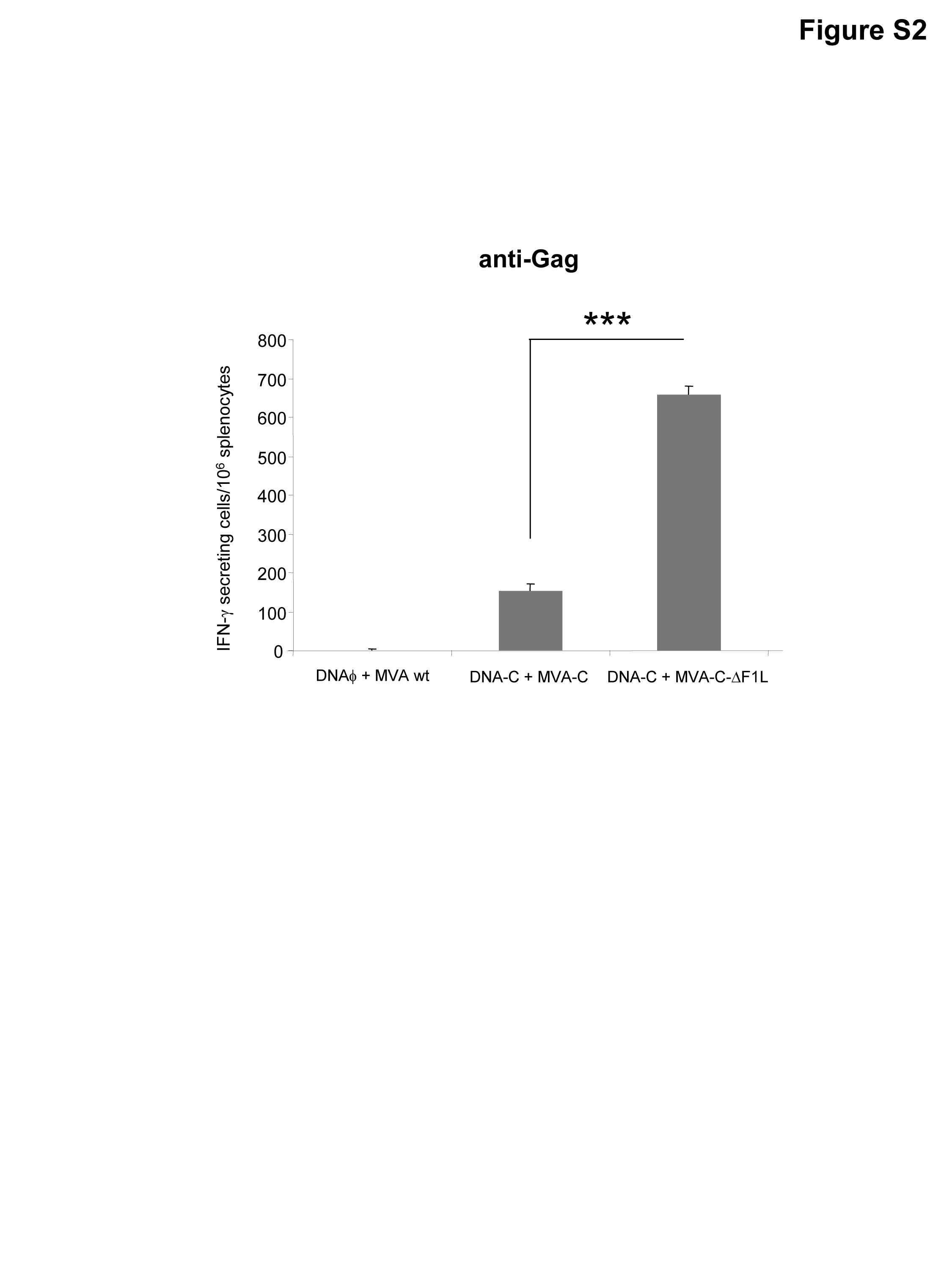

Supplement: Figure S2 — Adaptive Gag-specific T cell immune response elicited by F1L deletion mutant. Magnitude of the total Gag-specific T cell response was measured 10 days after the last immunization by ELISPOT assay following stimulation with the different HIV-1 peptide pools in 3 mice of each group (n = 3). Number of IFN-γ-secreting cells are represented. *** p<0.001. p value indicates significantly higher response compared to DNA-C/MVA-C immunization group. (TIF) [file pone.0048524.s002.tif]
